# Nasopharyngeal Carriage in Children After the Introduction of Generalized Infant Pneumococcal Conjugate Vaccine Immunization in Germany

**DOI:** 10.3389/fmed.2021.719481

**Published:** 2021-09-13

**Authors:** Markus A. Rose, Maren Laurenz, Ralf Sprenger, Matthias Imöhl, Mark van der Linden

**Affiliations:** ^1^Klinikum Stuttgart Olgahospital, Stuttgart, Germany; ^2^Pfizer Pharma GmbH, Berlin, Germany; ^3^Department of Medical Microbiology, National Reference Center for Streptococci, University Hospital (RWTH), Aachen, Germany; ^4^Laboratory Diagnostic Center, University Hospital (RWTH), Aachen, Germany

**Keywords:** nasopharyngeal carriage, children, Germany, post-PCV introduction, *S. pneumoniae*

## Abstract

Epidemiological data on nasopharyngeal (NP) bacterial carriage in children in Germany are scarce. We prospectively characterized NP colonization to evaluate the impact of pneumococcal immunization. We longitudinally collected NP swabs from 2-month-old infants (visit 1; V1) at eight representative pediatric offices 10/2008-06/2009. The second swabs were taken at age 9–12 months (V2); the third swab was taken 3–6 months after the booster vaccination at age 17–19 months (V3), and the fourth swab (V4) at age 59–61 months. Samples were broth enriched, cultured for bacteria, and isolates were serotyped. Demographic risk factors for colonization were evaluated. Among 242 vaccinees, bacterial NP carriage increased with age [from 27.2% (V1) to 70.1% (V4)]; leading isolates were *S. pneumoniae, H. influenzae, M. catarrhalis*, and *S. pyogenes*. Overall pneumococcal carriage increased [14.7% (V1), 31.5% (V2), 34.8% (V3), 42.2% (V4)], being even greater among day-care attendees. Serotype distribution changed during the study period, with vaccine serotypes declining. At visit 4, 10-valent pneumococcal conjugate vaccine (PCV10) serotypes were no longer among the NP flora, while some serotypes unique to 13-valent pneumococcal conjugate vaccine (PCV13; 3 and 19A) were found. In Germany, universal infant PCV immunization was associated with an almost complete eradication of PCV-serotypes and concomitant increase of non-PCV-serotypes, mainly 11A, 22F, and 23A.

## Introduction

Worldwide, young children are a major reservoir for pneumococci, with more than 700,000 infants dying from invasive pneumococcal disease each year (1). Non-invasive pneumococcal diseases like otitis media and pneumonia are widespread and significantly contribute to morbidity among children. For instance, almost every child experiences at least one episode of acute otitis media in the first 2 years of life (2). Thus, vaccine prevention of pneumococcal infections is a major public health issue (3, 4). Since 2006, for example, the inclusion in Europe of the 7-valent pneumococcal conjugate vaccine (PCV7) into national routine immunization programmes substantially reduced vaccine-serotype (VT) invasive pneumococcal disease (IPD) in all age groups (5–14). In parallel, a slight increase in non-vaccine serotype (NVT) IPD incidence was observed (15, 16). After transitioning from PCV7 to PCV13 in 2011, a further decrease in IPD was documented. Nonetheless, broader coverage pneumococcal conjugate vaccines will further block acquisition of nasopharyngeal (NP) carriage thereby opening new niches for colonization with NVT pneumococci or other bacteria, which prompts the World Health Organization (WHO) immunization surveillance recommendations to state that monitoring of NP colonization remains important in order to detect potential changes and to adapt vaccine recommendations if necessary (17).

The aim of this study was to assess the impact of pneumococcal conjugate vaccination on nasopharyngeal carriage in young children in Germany.

## Materials and Methods

### Demographics and Study Settings

Our prospective, multi-center, cohort, epidemiology study was designed to monitor in clinical practices NP carriage and any changes in pneumococcal serotype distribution among 2-to-61-month-old children. Our sample size calculation assumed that 10–80% of all preschool children are colonized with streptococci, depending on their age. To obtain a total number of 100 *S. pneumoniae*-positive NP samples, 240 children (960 NP swabs) were to be included in this study, assuming a 20% drop-out rate over the course of the study. A total of ten pediatric offices, representatively spread across Germany, were chosen as study sites, contributing 20–40 subjects each. Prior to inclusion, both parents/guardians provided written informed consent. Exclusion criteria were any dysplasia or impairment of the nasopharynx challenging swab sampling, known or suspected immune deficiency of the child, clinically relevant immune suppression, antibiotic treatment within 10 days or hospitalization within 2 weeks prior to inclusion in this study, a history of chronic infections, immunoglobulin therapy, haemato-oncological diseases, or the participation in other clinical trials.

The infants were enrolled regardless of their parents' intent to vaccinate them against pneumococci. Bacterial carriage was determined at a total of four visits by NP swabs with follow-up visits continued until the age of 5 years. Concomitantly, immunization status, medical history, use of antibiotics and relevant immunosuppressive drugs in the antecedent 3 months were documented.

**Figure 1:**
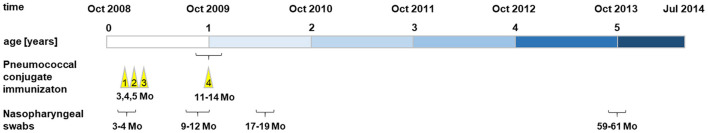
Study design.

Germany introduced routine PCV7 immunization of up to 2-year-old children in July 2006, with a primary immunization series at 2, 3, and 4 months of age, and a booster at the age of 11–14 months (Figure 1) (18). Accordingly, the first swab was taken prior to primary vaccination at an age of ~2 months (visit 1, V1). The second swab was taken 3–6 months after completed primary vaccination at approximately the age of 9–12 months (visit 2; V2); the third swab was taken 3–6 months after the booster vaccination at an approximate age of 17–19 months (visit 3; V3). To estimate long-term effects, the fourth and last swab was taken at an age of 59–61 months (visit 4; V4). The same time schedule applied to PCV unvaccinated children.

In the case of antibiotic therapy within the 10 days preceding a scheduled visit, swab sampling was postponed. During the study, the use of any medication could lead to postponement of a swab sampling, subject to the discretion of the investigator. Any administration of antibiotics and/or relevant immunosuppressive drugs within 3 months prior to nasopharyngeal swab sampling had to be documented. The study was approved by the ethical committees of the medical council of Westphalia-Lippe and the Westphalian Wilhelms-University of Münster (0887X1-4453); the human experimentation guidelines of Good Clinical Practice, the German Drug Act and the declarations of Helsinki/Hong Kong were followed in the conduct of clinical research. All guardians provided informed consent for their children. The study was initiated in October 2008, and to minimize seasonal effects on carriage, the timeframe for recruitment was 8 months, ending in June 2009.

### Nasopharyngeal Swab Sampling and Bacterial Work-Up

NP swab sampling was carried out according to the standard procedure of the WHO Pneumococcal Vaccine Trials Carriage Working Group (19). The nasopharyngeal swab was immediately placed into the dedicated test tube filled with Amies medium, which was labeled unambiguously and sent overnight by mail at ambient temperature to the German National Reference Center for Streptococci (Aachen).

NP swabs were inoculated directly from the Amies tube on Columbia blood agar, CNA agar, PVX-chocolate agar and *Haemophilus* chocolate agar. Identification of bacterial isolates was performed using routine microbiology techniques, including MALDI-TOF-MS (Bruker Biotyper, Germany; not used for identification of *S. pneumoniae*), focusing on *S. pneumonia, S. pyogenes, H. influenza*, and *M. catarrhalis*. Pneumococci were identified based on their colony appearance and biochemistry (alpha-haemolysis, sensitivity to optochin, and lysis by bile salts). Serotyping was performed with the Neufeld Quellung reaction using antisera from the Statens Serum Institute (Copenhagen, Denmark). Only one colony per plate was analyzed, unless clear morphological differences were observed. Multilocus sequence typing of pneumococcal isolates was performed as described previously (20). Briefly, internal fragments of the *aroE, gdh, gki, recP, spi, xpt*, and *ddl* genes were amplified by PCR from chromosomal DNA with the described primer pairs and sequenced using the Sanger method. A special allelic profile is provided by the alleles at each of the seven loci and their sequence type (ST) is defined. The allelic profiles were compared with each other and with other isolates in the pneumococcal MLST database using software available at http://pubmlst.org/spneumoniae/.

### Data Analysis

We performed a descriptive statistical analysis. For dichotomous data, the frequency of values was displayed including the 95% confidence interval (95%CI). Continuous variables were described with their statistical location parameters (average, standard deviation, median, minimum, and maximum). Changes of a dichotomous variable over time were described using the Armitage test for trends. Changes in serotype distribution were assessed using Fischer's exact test. The impact of environmental influences (e.g., tobacco smoke exposure, or day-care attendance) was investigated by multiple regression analysis.

Absolute and relative frequencies of total pneumococcal carriage were calculated based on all evaluable subjects, including the 95%CI. Additionally, we stratified by vaccine-serotype pneumococci to estimate any potential impact of PCVs on carriage. The analyses were performed separately for the different time points. Secondary target variables were intra-individual changes in nasopharyngeal carriage over time such as the number and the percentage of children changing pneumococcal carriage status (negative to positive) or changing serotype status (PCV serotype to non-PCV serotype). Serotype diversity was assessed by calculating the Simpson Diversity Index.

## Results

**Figure 2:**
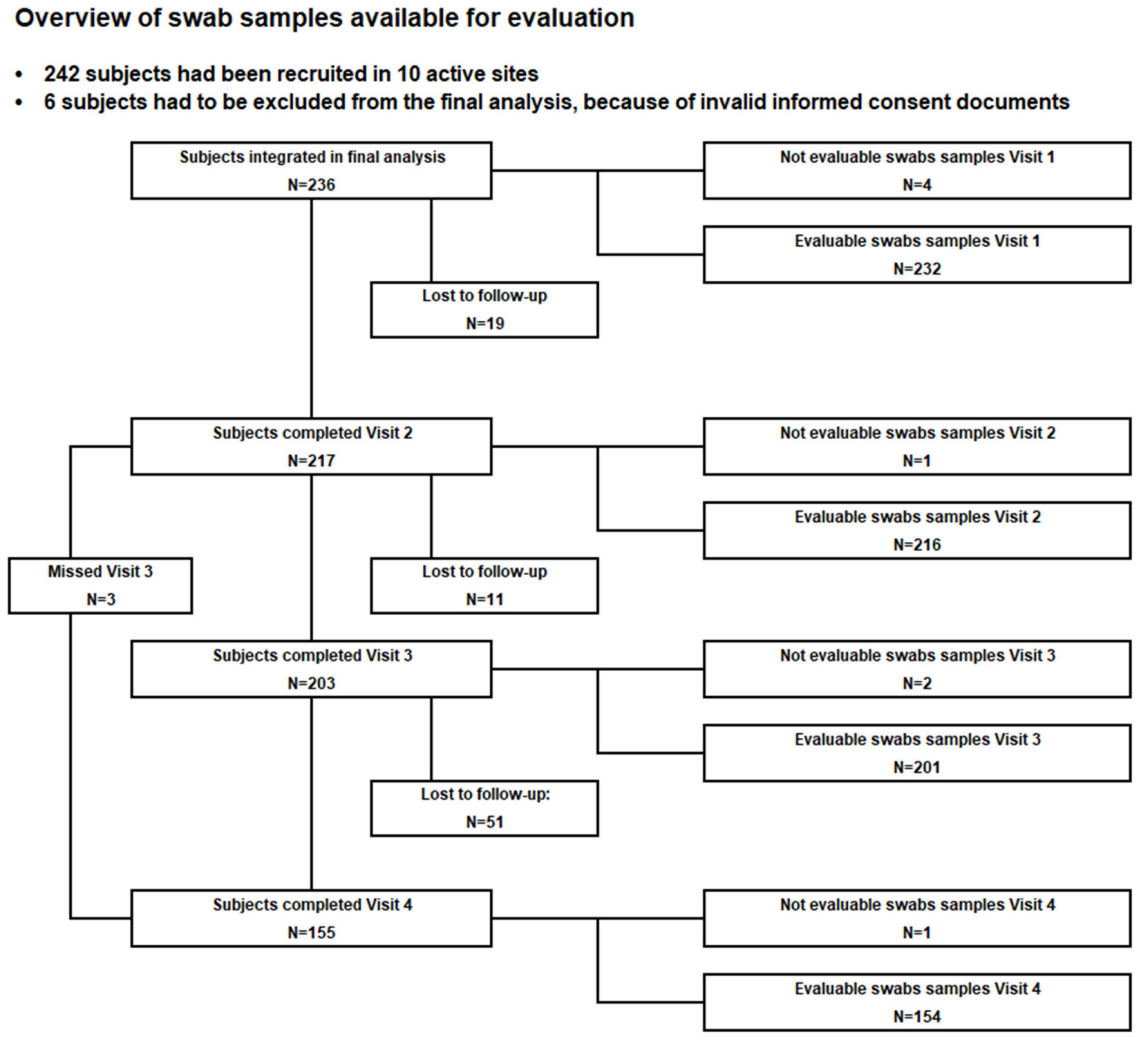
Course of the study.

Demographics and other baseline characteristics are described for all subjects available for final analysis, regardless of any treatment with antibiotics within the 10 days prior to study visits and regardless of any age deviation at study visits. Of the originally included 242 children, six were excluded from the final analysis due to incorrect or incomplete informed consent documentation. Thus, there were 236 subjects at visit 1, 217 subjects at visit 2, 203 subjects at visit 3, and 155 subjects at visit 4 (Figure 2). The study population was 54.2% male (128) and 45.8% female (108). The age distribution is presented in Table 1.

**Table 1:** Age distribution of the study population.

	**No. of subjects**	**Mean age (months)**	**SD**	**Median age (months)**	**Min**	**Max**
Visit 1	236	2.6	0.6	2.3	1.9	4.9
2	217	11.8	1.0	11.8	8.6	14.9
3	203	19.3	2.0	18.6	16.4	26.1
4	155	60.9	1.6	60.4	59.0	68.0

### Pneumococcal Immunization Status

At visit 1, at 2 months of age (prior to the primary vaccination series schedule), none of the 236 subjects had been vaccinated. At visit 2 (*n* = 217), at age 12 months of age (three to 6 months after completion of the primary vaccination schedule), 99.1% (*n* = 215) of the subjects were vaccinated (PCV7: 93.5%, PCV10: 6.5%), and five subjects had already been boosted (PCV7: *n* = 3; PCV10: *n* = 1; PCV13: *n* = 1). At visit 3, at age 19 months of age (3–6 months after the booster vaccination), all subjects (*n* = 203) had completed their primary immunization (PCV7: 93.1%, PCV10: 6.9%), and 84.2% (*n* = 189) had also received a booster dose (PCV7: 33.3%, *n* = 57; PCV10: 9.9%, *n* = 17; PCV13: 56.7%, *n* = 97). Of 155 subjects seen at visit 4 (age 60 months), 150 had received the booster (PCV7: 25.3%, *n* = 38; PCV10: 6.0%, *n* = 9; PCV13: 68.7%, *n* = 103; Supplementary Table 1). For analysis, subjects who had received at least one dose of PCV were regarded as vaccinated.

### Bacterial Isolates From NP Swabs Over the Course of the Study

NP swab bacterial isolates (*S. pneumoniae, H. influenzae, M. catarrhalis*, and *S. pyogenes*) are listed in Table 2. Of 232 subjects, 27.2% had bacterial isolates identified at visit 1. A single species was identified in 48 samples (20.7%), two species were identified in 13 samples (5.6%), and three species were found in two samples (0.9%).

**Table 2:** Nasopharyngeal colonizing bacterial spectrum in 2–60 month-old children in the course of the study – number of colonized subjects.

**Number of colonized subjects**											**% of subjects *S. pneumoniae* positive**
**Visit 1**	**Total (** ***n*** **=** **232)**										**14.7**
**Number of colonizing bacteria**	**0**		**1**		**2**		**3**		**All subjects any bacteria**		
	***n***	**%**	***n***	**%**	***n***	**%**	***n***	**%**	***n***	**%**	
Pathogens identified	169	72.8	48	20.7	13	5.6	2	0.9	63	27.2	
**Visit 2**	**Total (** ***n*** **=** **216)**										**31.5**
Pathogens identified	97	44.9	80	70.0	34	15.7	5	2.3	119	55.1	
**Visit 3**	**Total (** ***n*** **=** **201)**										**34.8**
Pathogens identified	85	42.3	56	27.9	47	23.4	13	6.5	116	57.7	
**Visit 4**	**Total (** ***n*** **=** **154)**										**42.2**
Pathogens identified	46	29.9	56	36.4	41	26.6	11	7.1	108	70.1	

*Percentages were calculated in relation to all subjects providing evaluable swab samples at the respective visits. As a consequence, percentages add up to more than 100. %, percentage; Spn, Streptococcus pneumonia*.

At visit 2, we identified 163 bacterial isolates in 119 (55.1%) subjects. One species was identified in 80 samples (37.0%), two species in 34 samples (15.7%) and three species in five samples (2.3%). At visit 3, we detected 189 bacterial isolates in 116 (57.7%) subjects. One species was identified in 56 samples (27.9%), two species in 47 samples (23.4 %) and three species in 13 samples (6.5%). At visit 4, we found 171 bacterial isolates in 108 (70.1%) subjects. One species was identified in 56 samples (36.4%), two species in 41 samples (26.6%) and three species in 11 samples (7.1%).

Overall, we observed bacterial carriage to increase with age. In general, children carried between one and three species simultaneously. At visit 1 (age about 3 months), 27.2% of our subjects had any colonization, at visit 2 (age 1 year), 55.1%, at visit 3 (age 20 months), 57.7%, and finally 70.1% at visit 4 (age 5 years). Most prevalent potential pathogens were *S. pneumonia*e, *M. catarrhalis, H. influenzae*, and *S. pyogenes*. Details on bacterial species findings and the combinations of different species found are provided in Supplementary Table 2.

**Figure 3:**
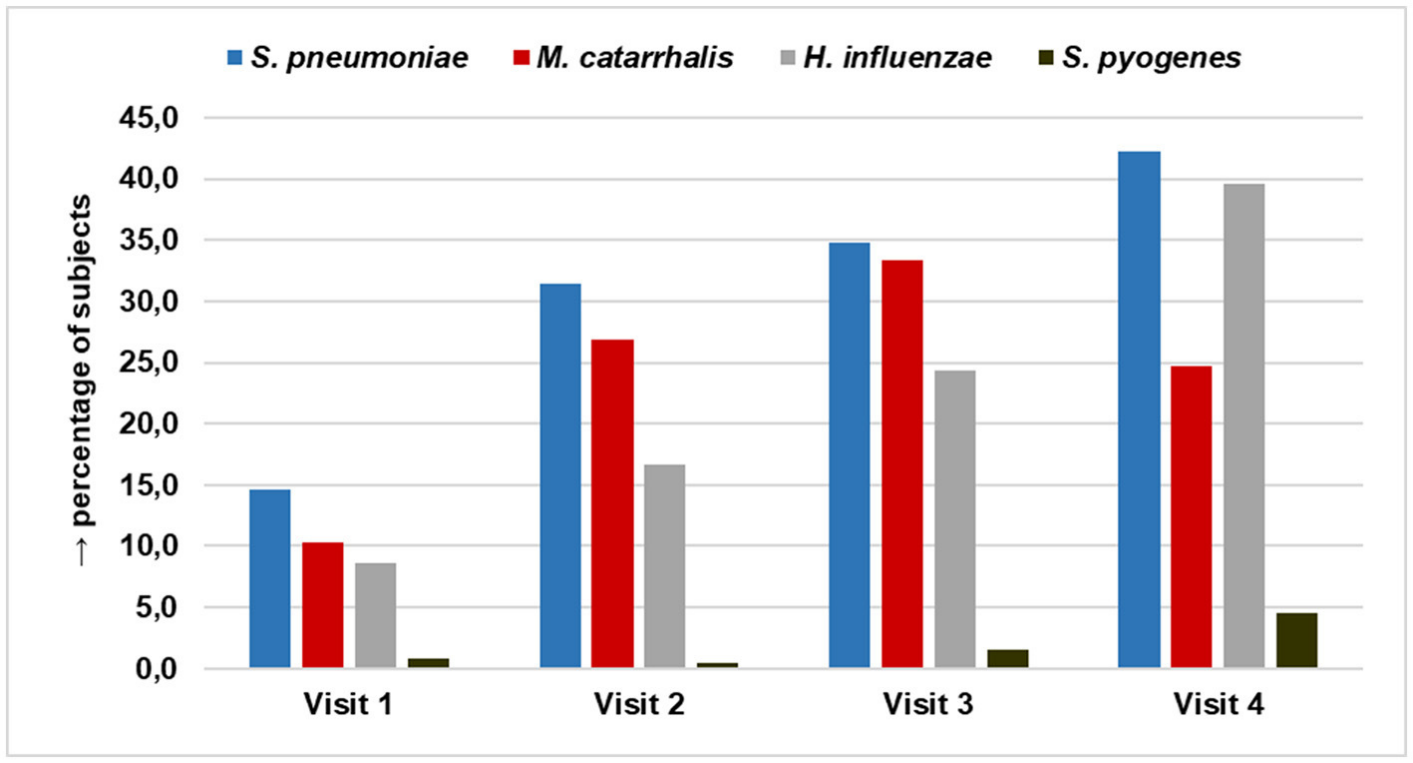
Percentage of nasopharyngeal colonization by *S. pneumonia, M. catarrhalis, H. influenza*, and *S. pyogenes* among 2–60 month old children in the course of the study.

Among all species identified at the respective time points, *S. pneumoniae* was most prominent over the entire course of the study (Table 3; Figure 3). Overall, at visit 1, 34 of 232 subjects [14.7% (95% CI: 10.1–19.2)] showed pneumococcal carriage, at visit 2, 68 of 216 subjects [31.5% (25.3–37.7)], and 70 of 201 subjects [34.8% (28.2–41.4)] at visit 3. At visit 4, pneumococcal carriage was observed in 65 of 154 subjects [42.2% (34.4–50.0)].

**Table 3:** Nasopharyngeal colonizing bacterial spectrum in 2–60 month old children in the course of the study - number of specific strains per visit.

	**Visit 1**		**Visit 2**		**Visit 3**		**Visit 4**	
	***n***	**%**	***n***	**%**	***n***	**%**	***n***	**%**
**Subjects**	***n*** **=** **232**		***n*** **=** **216**		***n*** **=** **201**		***n*** **=** **154**	
*S. pneumoniae*	34	14.7	68	31.5	70	34.8	65	42.2
*M. catarrhalis*	24	10.3	58	26.9	67	33.3	38	24.7
*H. influenzae*	20	8.6	36	16.7	49	24.4	61	39.6
*S. pyogenes*	2	0.9	1	0.5	3	1.5	7	4.5

Although the rate of pneumococcal carriage increased with age, pneumococcus continued to represent about 40% of all identified bacteria throughout the four visits. This trend is underlined by the logistic model calculations for *S. pneumoniae* carriage at visit 2 and visit 3, showing a significantly lower risk for carrying *S. pneumoniae* in comparison with other pathogens with increasing age of subjects [V2 odds-ratio: 0.93 (95% CI: 0.86–0.98); V3 odds-ratio: 0.96 (0.93–0.98)]. For details refer to Supplementary Table 3.

### *S. pneumoniae* Serotype Distribution and PCV-Coverage

At visit 1, the most prominent serotypes identified were 11A (23.5%), 6A, 9N (11.8% each), and 15B (8.8%). Two different serotypes covered by PCV7 (6B, 19F) were detected in a total of four pneumococcal isolates (11.8%), three serotypes (1, 6B, 19F) covered by PCV10 were detected in six (17.7%) isolates overall. Five serotypes (1, 6A, 6B, 19A, 19F) covered by PCV13 were detected in a total of 11 (32.4%) isolates. A total of 16 pneumococcal serotypes (47.1%) exclusively covered by the 23 valent pneumococcal polysaccharide vaccine (PPV23-/nonPCV13-serotypes) were identified at this early time (Table 4).

**Table 4:** *S. pneumoniae* serotype distribution in the course of the study.

**Serotype**	**Visit 1**		**Visit 2**		**Visit 3**		**Visit 4**	
	***n***	**%**	***n***	**%**	***n***	**%**	***n***	**%**
6B	2	5.9	0	0.0	1	1.4	0	0.0
9V	0	0.0	1	1.5	0	0.0	0	0.0
14	0	0.0	0	0.0	1	1.4	0	0.0
18C	0	0.0	1	1.5	0	0.0	0	0.0
19F	2	5.9	2	2.9	1	1.4	0	0.0
23F	0	0.0	4	5.9	2	2.9	0	0.0
**PCV7**	**4**	**11.8**	**8**	**11.8**	**5**	**7.1**	**0**	**0.0**
1	2	5.9	0	0.0	0	0.0	0	0.0
7F	0	0.0	1	1.5	0	0.0	0	0.0
3	0	0.0	1	1.5	3	4.3	4	6.2
6A	4	11.8	6	8.8	3	4.3	0	0.0
19A	1	2.9	5	7.4	6	8.6	2	3.1
**PCV13**	**11**	**32.4**	**21**	**30.9**	**17**	**24.3**	**6**	**9.2**
8	0	0.0	1	1.5	0	0.0	1	1.5
9N	4	11.8	3	4.4	0	0.0	2	3.1
10A	0	0.0	3	4.4	1	1.4	1	1.5
11A	8	23.5	9	13.2	5	7.1	7	10.8
15B	3	8.8	6	8.8	2	2.9	1	1.5
17F	0	0.0	0	0.0	1	1.4	0	0.0
22F	1	2.9	2	2.9	3	4.3	4	6.2
33F	0	0.0	0	0.0	2	2.9	3	4.6
**PPV23**	**23**	**67.6**	**39**	**57.4**	**28**	**40.0**	**25**	**38.5**
6C	0	0.0	2	2.9	7	10.0	0	0.0
10B	0	0.0	0	0.0	0	0.0	1	1.5
15A	0	0.0	0	0.0	2	2.9	2	3.1
15C	2	5.9	3	4.4	10	14.3	3	4.6
16F	0	0.0	1	1.5	1	1.4	0	0.0
21	0	0.0	0	0.0	1	1.4	3	4.6
23A	1	2.9	1	1.5	5	7.1	5	7.7
23B	1	2.9	3	4.4	3	4.3	2	3.1
24F	0	0.0	0	0.0	2	2.9	6	9.2
28A	0	0.0	1	1.5	0	0.0	0	0.0
28F	0	0.0	1	1.5	0	0.0	1	1.5
31	0	0.0	1	1.5	0	0.0	3	4.6
34	1	2.9	0	0.0	0	0.0	1	1.5
35B	0	0.0	2	2.9	1	1.4	3	4.6
35C	0	0.0	1	1.5	0	0.0	0	0.0
35D	0	0.0	0	0.0	1	1.4	0	0.0
35F	1	2.9	7	10.3	4	5.7	3	4.6
38	1	2.9	0	0.0	0	0.0	2	3.1
NT	0	0.0	0	0.0	2	2.9	5	7.7
**All**	**34**	**100.0**	**68**	**100.0**	**70**	**100.0**	**65**	**100.0**

*Orange, PCV7 serotypes; blue, additional PCV13 serotypes; yellow, Serotypes solely included in PPV23*.

At visit 2, the most prominent serotypes identified were 11A (13.2%), 35F (10.3%), 6A, 15B (8.8% each), 19A (7.4%), and 23F (5.9%). Four serotypes (9V, 18C, 19F, 23F) covered by PCV7 were detected in a total of eight isolates (11.8%). Five serotypes (7F, 9V, 18C, 19F, 23F) covered by PCV10 were detected in overall nine isolates (13.2%). Eight serotypes (3, 6A, 7F, 9V, 18C, 19A, 19F, 23F) covered by PCV13 were detected in a total of 21 (30.9%) isolates. A total of 24 PPV23-/nonPCV13-serotypes (35.3%) was identified at visit 2.

At visit 3, the most prominent serotypes identified were 15C (14.3%), 6C (10.0%), 19A (8.6%), 11A, 23A (7.1% each), and 35F (5.7%). Four serotypes (6B, 14, 19F, 23F) covered by PCV7 were detected in a total of five isolates (7.1%). Four serotypes (6B, 14, 19F, 23F) covered by PCV10 were detected in overall five isolates (7.1%). Seven serotypes (3, 6A, 6B, 14, 19A, 19F, 23F) covered by PCV13 were detected in a total of 17 (24.3%) isolates. We detected 14 PPV23-/nonPCV13-serotypes (20.0%) at visit 3.

At visit 4, the most prominent serotypes identified were 11A (10.8%), 24F (9.2%), 23A and NT (7.7% each), 3 and 22F (6.2% each). No PCV7 or PCV10 serotypes were detected at visit 4. Only two PCV13 serotypes (3 and 19A) were found in a total of six isolates (9.2%). This almost complete eradication of vaccine serotypes was accompanied by an increase of *H. influenzae* (mainly non-typeable strains) and of non-vaccine serotype pneumococci, mainly 11A (10.8%), 24F (9.2%), and 23A (7.7%) at visit 4. At this final visit, 19 PPV23-/nonPCV13-serotypes (29.2%) could be identified in the nasopharynx.

The pneumococcal serotype diversity calculated by the Simpson Diversity Index (SDI) was 0.92 at visit 1, 0.95 at visit 2, 0.97 at visit 3 and 0.96 at visit 4. Concerning co-carriage of *S. pneumoniae* and *H. influenzae* SDIs were 0.96 and 0.97 for carriage of *S. pneumoniae* with *H. influenzae*, vs. carriage of *S. pneumoniae* without *H. influenzae*. Serotypes 3, 10A, 19A, 31, and 33F were more prevalent among subjects with co-carriage of *S. pneumoniae* and *H. influenzae*, whereas serotypes 6A, 6C, 19F, and 23F were more prevalent among subjects carrying *S. pneumonia* without *H. influenza*, however, none of these differences reached statistical significance (Supplementary Figure 1).

### Impact of Vaccination Status on *S. pneumoniae* Serotypes During Visits

At visit 2, we found no relevant impact of the primary immunization (PCV7 or PCV10) on the serotype distribution. At visit 3 (shortly after the booster), an initial reduction of vaccine serotypes could be observed. Overall, the percentage of subjects carrying PCV7-VT pneumococci decreased during this study from 3.7% at the time of completed primary immunization (visit 2) to 2.5% shortly after completed booster vaccination (visit 3), and to zero at visit 4. Four years after booster vaccination (visit 4), there were no PCV7 serotypes or serotypes 1, 5, or 7F, and only a few of serotypes 3 (*n* = 4), or 19A (*n* = 2) were found, while overall pneumococcal carriage increased with age. At visit 3, only twelve subjects carried serotypes 3 (*n* = 3), 6A (*n* = 3), or 19A (*n* = 6) despite a PCV13 booster (Supplementary Table 4).

### Changes in *S. pneumoniae* Serotype Carriage Status Between Visits

In the course of our study, 58.6% (*n* = 136) of our subjects were positive for *S. pneumoniae* at any visit. Among them, 44.1% (*n* = 60) carried pneumococci only at one visit, and six subjects (4.4%) were positive at all four visits. Fifty-three (39.0%) acquired pneumococci and stayed colonized, 75 (55.1%) acquired *S. pneumoniae* and then eliminated them at a later visit. Of these, 13 (9.6%) reacquired pneumococci, of which one individual eliminated them once more. Eight individuals (5.7%) did not show up at one or more follow-up visits (Supplementary Table 4). Sixteen subjects carried the same or highly related serotype at different visits. Of these, twelve also had the same sequence type, and four subjects had the same sequence type, with highly related serotypes [6B/6C, 15B/15C (2x), 35B/35D]. One subject (0619) carried isolates of serotypes 1 and 19F, both with sequence type 179. Another subject (0221) carried serotype 9N at visits 1 and 2, and non-encapsulated pneumococci (NT) at visit 3, with all three isolates of sequence type 66 (Supplementary Tables 4, 5).

### Environmental Influences on *S. pneumoniae* Carriage

At visits 1 and 2 (mean ages 2.6 and 11.8 months, respectively), few subjects were attending day-care centers (V1: 0.4%; V2: 2.3%), and none of these children who were attending day care tested positive for *S. pneumoniae*. For details of visit 1 and 2, refer to Supplementary Table 5.

Data at visit 3 (mean age 19.3 months), and visit 4 (mean age 60.9 months) are presented in Table 5. At visit 3 and visit 4, pneumococcal carriage of subjects in day-care was at comparable levels (V3: 45.8%, V4: 43.0%), and noticeably higher than those of children who were not cared for in communal facilities (V3: 33.3%, V4: 0.0%). At visit 4, a few subjects were not in day-care; none tested positive for *S. pneumoniae*.

**Table 5:** Day-care and *S. pneumoniae* colonization.

**Subjects in day-care**	***S. pneumoniae*** **isolates**					
	**Positive**		**Negative**		**Total (** ***n*** **=** **201)**	
**Visit 3**	***n***	**(%)^a^**	***n***	**(%)**	***n***	**%**
No	59	33.3	118	66.7	177	88.1
Yes	11	45.8	13	54.2	24	11.9
**Visit 4**					**Total (** ***n*** **=** **154)**	
No	0	0.0	3	100	3	1.9
Yes	65	43.0	86	57.0	151	98.1

*N = total number of subjects providing evaluable swab samples at the respective visit. (%)*

a *: incidence rate in % = percentage of cases based on the total number of subjects with respective day-care status*.

Irrespective of the vaccinees being in day-care or not, pneumococcal carriage in subjects without siblings in day-care increased from 0.9% at visit 1 to 15.7% at visit 2 (after completion of primary immunization), to 24.7% at visit 3 (shortly after completion of booster vaccination), and to 55.3% at visit 4 (4 years after completion of booster vaccination). As expected, pneumococcal colonization in subjects with siblings in day-care was distinctly higher at the start of the study, 27.4% at visit 1, and increased until visit 2 (48.0%) at the time of completed primary immunization. Remarkably, and in contrast to subjects without siblings in day-care, carriage in these subjects decreased at visit 3 (44.1%) and visit 4 (38.6%).

Domestic tobacco smoke exposure is a well-known risk factor for otitis media and pneumonia, and results from the current study are summarized in Tables 6, 7. Constantly over the whole study, one of three infants was exposed to domestic tobacco smoke. At visits 1–3, pneumococcal colonization did not differ depending on tobacco smoke exposure. By the time of visit 4, subjects without smoke exposure showed an increase to 47.3% in pneumococcal colonization, while rates in subjects with smoke exposure remained unchanged at 29.5%.

**Table 6:** Domestic tobacco smoke exposure.

**Domestic tobacco smoke exposure**	**Visit**							
	**1**		**2**		**3**		**4**	
	***n***	**%**	***N***	**%**	***n***	**%**	***n***	**%**
No	160	67.8	152	70.0	142	70.0	111	71.6
Yes	76	32.2	65	30.0	61	30.0	44	28.4
All	236	100	217	100	203	100	155	100

**Table 7:** Domestic tobacco smoke exposure and pneumococcal colonization.

**Domestic tobacco smoke exposure**		***S. pneumoniae*** **positive**		***S. pneumoniae*** **negative**		**All**	
		***n***	**%**	***n***	**%**	***n***	**%**
**Visit**							
1	No	22	13.9	136	86.1	158	100
	Yes	12	16.2	62	83.8	74	100
2	No	48	31.8	103	68.2	151	100
	Yes	20	30.8	45	69.2	65	100
3	No	51	36.2	90	63.8	141	100
	Yes	19	31.7	41	68.3	60	100
4	No	52	47.3	58	52.7	110	100
	Yes	13	29.5	31	70.5	44	100

## Discussion

The objective of this study was to analyse the impact of the German infant immunization programme with PCVs on the NP carriage of healthy children up to 5 years of age. In Germany, PCV7 was introduced in July 2006 as part of the National Immunization Programme with a 3 + 1 schedule and was replaced by higher-valent vaccines in 2009 (PCV10 in April, PCV13 in December), with PCV13 dominating the market (currently > 95%). Following systematic PCV immunization, a marked decrease of IPD was observed in all age groups. As recruitment for this study ended in June 2009, most of the subjects received primary immunization with PCV7, and some with PCV10. For the booster immunization (October 2009 to June 2010), mainly PCV10 and PCV13 were available; nonetheless, 25.3% still completed their PCV immunization with PCV7, a phenomenon often observed during transitions.

In countries without PCV immunization, average colonization rates in children aged <5 years are about 65% (95% CI: 49.8–76.1%) in low-income countries (e.g., Bangladesh, Tanzania), and about 48% (95% CI: 44.7–50.8%) for lower-middle income countries (Fiji, Gaza strip, India, Indonesia, and Vietnam).

Our time-series prevalence survey assessed NP colonization in young children after PCV implementation in Germany, with an observed age-dependent increase of NP *S. pneumoniae* colonization rates from 14.7% (2 month-old infants) to 42.2% in 5-year olds.

In Israel, PCV7 and PCV13 were introduced into the Israeli National Immunization plan in July 2009 and November 2010, respectively. A large prospective, population-based, active surveillance trial assessed pneumococcal conjugate vaccine (PCV) uptake and dynamics in serotype-specific pneumococcal nasopharyngeal (NP) carriage in <5 year old children during the subsequent 5 years. Among 10,702 vaccinees, a regular increase of pneumococcal carriage with age was observed among healthy children: from 27% (0–3 month-olds), 52% (6–12 month olds), 58% (12–23 month olds) to 60% (24–35 month olds) (10). The same group demonstrated a mild decrease of *S. pneumoniae* colonization in Jewish <5 year old children from 45.7% (early PCV7-era) to 42% (PCV13-era). Remarkably, the impact of PCVs was in parallel stronger in Bedouin children, underlining the relevance of socio-economic factors in pneumococcal disease.

Remarkably, in Norway as a typical high-income country, up to 80% of children in day care centers were colonized with pneumococci in pre-PCV times, underlining the big impact of crowding on nasopharyngeal colonization (21). In 2015, 4 years after the introduction of PCV13 in Norway, pneumococcal carriage levels were 38% lower (22). A survey performed 3 years after PCV13 introduction (from July 2010 through June 2013) in 2,048 U.S. children (mean age 27 months) found a NP pneumococcal carriage rate of 32% (23). Another cross-sectional study on 1,250 healthy Italian children (age 3–59 months) 1 year after the introduction of PCV13 demonstrated an average pneumococcal NP colonization rate of 27% (24). Cross-sectional studies performed among children up to 6 years of age at day-care centers in Portugal, before and after PCV13 introduction showed a persistently high carriage rate of over 60%, but an overall reduction in vaccine serotypes (25).

We also found significant pneumococcal serotype changes among children in Germany after PCV immunization. While the overall rate of pneumococcal NP carriage increased during our 5-year study period from initially 14.7 to 42.2% at visit 4, no PCV7 serotypes or serotypes 1, 5, 6A or 7F and only six of serotypes 3 and 19A were isolated at visit 4, highlighting the ability of PCVs to block acquisition of vaccine serotypes. This almost complete eradication of vaccine serotypes was accompanied by an increase of *H. influenzae* (mainly non-typeable strains) and of non-vaccine serotype pneumococci, mainly 11A (10.8%), 24F (9.2%), and NT and 23A (7.7% each) at visit 4. Among the later ones, at least serotype 11A is included in PPV23.

Similar trends were observed in Cyprus and France, where serotype 19A remained the most prominent VT serotype, and frequent NVTs were 6C, 11A, 15A, 15B/C, 23A, and 35B (13, 26). In Belgium, direct effects on carriage of serotypes included or not included in different PCVs were immediately noticed after switching from one PCV formulation to the other (27, 28). We observed a consistent decline of PCV13 serotypes until visit 4, suggesting that a PCV13 boosting after primary PCV7 or PCV10 series is able to reduce PCV13 serotype colonization.

After vaccination, serotype diversity generally increased, depending on the country, time since introduction of higher-valent PCVs and the PCVs used. In our survey, we observed replacement in carriage in parallel with an overall marked reduction in pneumococcal disease in Germany, implying that replacing serotypes tend to be less invasive strains with a lower disease potential (7, 9). This is in line with observations from the literature that upcoming non-PCV13 serotypes tend to be less invasive (i.e., with low case-carrier ratios), and that newly emerging serotypes tend to be less aggressive than were 19A and 7F, for instance, after the introduction of PCV7.

Our MLST data reveal that several individuals carried the same pneumococcal clone over longer time periods (as judged by sequence type and serotype). Also, switching between highly related serotypes (6B/6C, 15B/15C, 35F/35D), with the sequence type staying the same, was observed on four occasions. One individual (0221) carried the same clone (ST66) over three visits, showing an eventual loss of the 9N capsule, possibly due to mutations or deletions in the capsular genes. Our findings seem to be illustrative of the active genetic exchanges going on among pneumococci and other viridans streptococci during nasopharyngeal carriage (29).

The diversity of the serotype distribution was high and changed little for all four sampling time points. Also, a very similar and high serotype diversity was observed for *S. pneumoniae* carriage with or without *H. influenzae*, which is in contrast to findings of Lewnard et al., who found reduced serotype diversity in children co-colonized with pneumococcus and non-typeable *H. influenzae* (NTHi) (30). Even though we found that serotypes 3, 10A, 19A, 31 and 33F were more prevalent among subjects co-colonized with *S. pneumoniae* and *H. influenzae*, whereas serotypes 6A, 6C, 19F and 23F were more prevalent among subject carrying *S. pneumoniae* without *H. influenzae*, the differences were not statistically significant. Therefore, our findings cannot confirm the findings of the study of Lewnard et al., who report that NTHi colonization was more prevalent among children carrying pneumococcal serotypes with greater capsular thickness, neutrophil resistance, and metabolic efficiency (30).

Even when we focus on non-PCV13 serotypes causing invasive pneumococcal disease, serotypes 8, 15A, 22F and 24F are predominant in countries using PCV13 to date (31). Data generated at the German National Reference Center for Streptococci also confirmed a significant decrease in PCV13-serotype IPD, while a diversity of non-PCV13 serotypes emerged in some age groups, and serotype 3 increased mainly in adults. A significant increase in pneumococcal serotypes 15A and 23B was observed for all age groups as well (31, 32).

Our multivariable analysis revealed day-care attendance to be associated with higher *S. pneumoniae* carriage rates. At visits 3 and 4, when the majority of infants attended day-care, incidence rates for pneumococcal carriage were significantly higher in day-care children than for children who were cared for at home (V3: 45.8 vs. 33.3%, V4: 43.0 vs. 0.0%, respectively). Irrespective of the vaccines being in day care or not, having siblings in day-care favored pneumococcal carriage, increasing from 27.4% at the study start to 48.0% at visit 2, compared to 0.9 and 15.7% in subjects without siblings in day-care. This is in line with data from Italy, where in a similar age group (3–59 months), having siblings and day-care attendance as well were associated with higher *S. pneumoniae* carriage (24). Also in a study performed in the USA, having siblings and attending day-care favored pneumococcal colonization (23). Remarkably, in our study, 5 year old children without siblings in day-care had higher pneumococcal colonization rates than those with siblings in day care (55.3 vs. 38.6%).

Host factors affecting colonization may modify immune defenses against a pathogen, but may also alter the composition of the resident microbiome. Colonizing bacteria must be able to overcome host defenses and to compete effectively with the resident microbiota. Thus, the host immune responses do not only influence the pathogen directly, but also the microbiome at the site of colonization as a whole, favoring resistance and resilience to pathogen invasion (33). Pneumococcal nasopharyngeal colonization usually lasts <6 months, inducing specific antibody responses directed at the carbohydrate capsule of the bacterium, facilitating pneumococcal clearance (34). Recent studies indicate that cell-mediated immunity, especially CD4+ cells producing cytokines of the interleukin-17 family, seem to be important for the clearance of colonizing pneumococci and protection from subsequent infections (35). Changes in the airway microbiome will also influence whether an invading pneumococcal strain will overcome the resident microflora and remain as an established colonizer, or will be cleared. Even competition between colonizing pneumococcal strains, other resident bacteria and the invading pneumococci via pathogen factors such as bacteriocins impacts on the acquisition of new strains (36). In conclusion, our observed reduced pneumococcal colonization of children in day-care compared to children cared-for at home might be due to the permanent stimulation of the immune system from the contact with wild-type pneumococci.

While domestic tobacco smoke exposure is a well-known risk factor for IPD, significant differences in carriage were not detected until visit 3 (age about 20 months). Of interest, our 5-year-old subjects without domestic smoke exposure showed an increase in pneumococcal colonization at visit 4, while rates in tobacco-smoke-exposed subjects remained stable at about 30%. It's well-known that active or passive cigarette smoking is a strong factor for invasive pneumococcal disease (37) and that an exposure to tobacco smoke also favors pneumococcal carriage in children (38). Since our observation is not in accordance with biomedical plausibility, a reasonable explanation might be the higher drop-out rate of children from smokers' households (40.5% from V1 to V4) as compared to non-smokers (25.3%), resulting in a bias of our results. None of our subjects contracted IPD in the course of our study.

**Figure 4:**
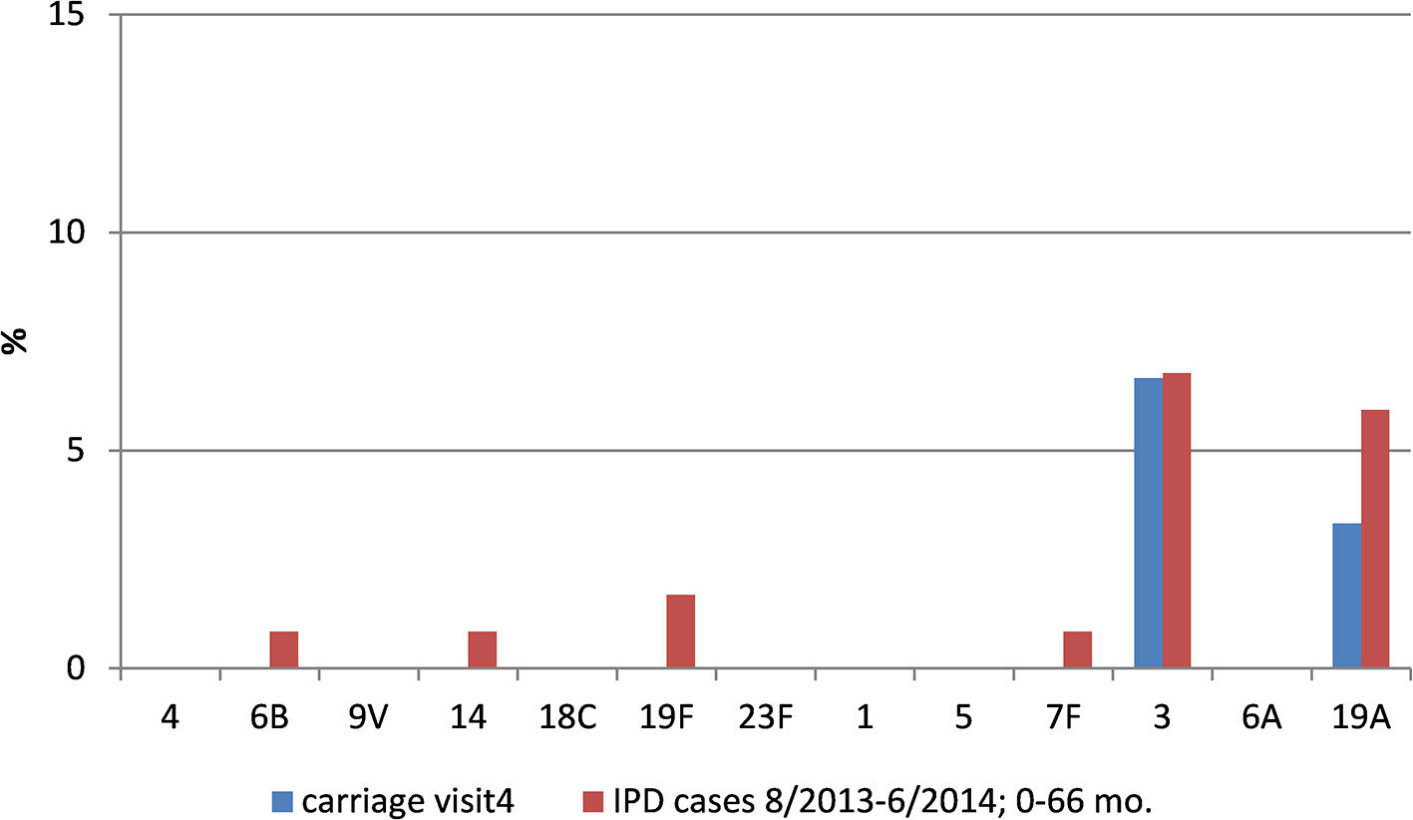
Pneumococcal vaccine-type carriage in our study population and IPD data from comparable age groups.

**Figure 5:**
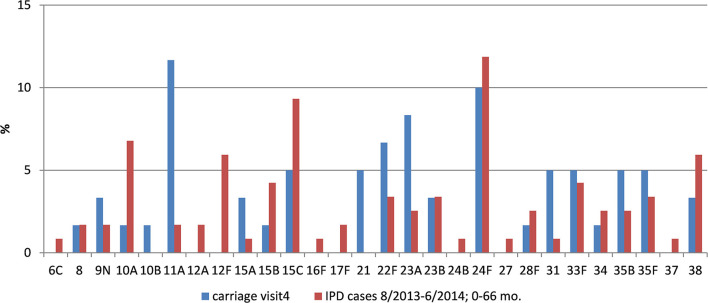
Pneumococcal non-vaccine-type carriage in our study population and IPD data from comparable age groups.

To put our colonization data into context with IPD, we used age- and time-matched data from the German National Reference Center for Streptococci (Figures 4, 5). Our data confirm the reduction of IPD in parallel with the mucosal decolonization of potentially-invasive pneumococcal serotypes.

However, despite significant direct and indirect protection, vaccine serotypes such as 3 and 19A are still circulating, being particularly important causes of IPD in older age groups. Data from the literature indicate that herd protection requires several years after universal infant immunization, especially in countries with lower pediatric vaccine uptake. This underlines the importance of national immunization programmes and continuous pneumococcal serotype surveillance.

It must be kept in mind that, from the public health perspective, pneumococcal disease mainly presents as non-invasive acute otitis media (AOM) and descending pneumonia. Facing the diagnostic and ethical challenges to obtain appropriate cultures for testing, NP colonization studies may serve as a surrogate to monitor serotype evolution in non-invasive disease, as mucosal pneumococcal disease results from direct infection. For instance, surveillance data from France did not establish an increase of NVT in AOM cases after the introduction of PCV13 (5).

The major strength of our study lies in it being a long-term, multicentre prospective surveillance of PCV impact on NP carriage representatively performed all over Germany. The repeated obtainment of NP cultures enables better appreciation of carriage dynamics post-PCV introduction. A limitation in our study is the lack of carriage data before the introduction of PCV into the national vaccination program. Thus, we could only estimate the impact of herd protection from the literature. Facing the impressive burden of pneumococcal disease reduction due to PCVs, it would have been unethical to run an unvaccinated control group. Secondly, since this was a longitudinal study, the results provide only a snapshot in time of pneumococcal colonization in this population. However, our study encompasses data from more than 5 years.

## Conclusions

This prospective, multi-center, longitudinal study provides data on PCVs' impact on NP carriage in young children. Our active survey demonstrates a decline in pneumococcal colonization, facilitated by a substantial decrease of VT pneumococci (even 19A), and an increase of non-VT pneumococci in Germany.

## Data Availability Statement

The original contributions presented in the study are included in the article/Supplementary Material, further inquiries can be directed to the corresponding author/s.

## Ethics Statement

The studies involving human participants were reviewed and approved by ethical committees of the medical council of Westphalia-Lippe and the Westphalian Wilhelms-University of Münster (0887X1-4453). Written informed consent to participate in this study was provided by the participants' legal guardian/next of kin.

## Author Contributions

MR, ML, RS, MI, and MvdL conceived the study. Microbiological analyses were performed by MvdL and MI. MR and MvdL performed the data analyses and wrote the manuscript. All authors have read and approved the manuscript.

## Funding

This research was sponsored by Pfizer.

## Conflict of Interest

MR, MI, and MvdL received financial support for research projects, traveling grants, and speakers' fees for scientific lectures from vaccine producing and distributing pharmaceutical companies. ML and RS are employees of Pfizer GmbH, Germany.

## Publisher's Note

All claims expressed in this article are solely those of the authors and do not necessarily represent those of their affiliated organizations, or those of the publisher, the editors and the reviewers. Any product that may be evaluated in this article, or claim that may be made by its manufacturer, is not guaranteed or endorsed by the publisher.
