# Supplementary material for: Nasopharyngeal Carriage in Children After the Introduction of Generalized Infant Pneumococcal Conjugate Vaccine Immunization in Germany
Source: Front Med (Lausanne). 2021 Sep 13;8:719481. doi: 10.3389/fmed.2021.719481 (PMC8473806; doi:10.3389/fmed.2021.719481)
Supplement: Supplementary file 1 [file Data_Sheet_1.docx]

Supplemental Table 1: Pneumococcal immunization status and colonization

| **Visit** | **Pneumococcal primary immunization** | ***S. pneumoniae*** | | | | | | **all** | |
| --- | --- | --- | --- | --- | --- | --- | --- | --- | --- |
|  |  | **unknown** | | **negative** | | **positive** | |  |  |
|  |  | **no. of subjects** | **%** | **no. of subjects** | **%** | **no. of subjects** | **%** | **no. of subjects** | **%** |
| **1** | **no** | 4 | 1.7 | 198 | 83.9 | 34 | 14.4 | 236 | 100 |
|  | **yes** | 0 | 0 | 0 | 0 | 0 | 0 | 0 | 0 |
|  | **all** | 4 | 1.7 | 198 | 83.9 | 34 | 14.4 | 236 | 100 |
| **2** | **no** | 0 | 0 | 2 | 100 | 0 | 0 | 2 | 100 |
|  | **yes** | 1 | 0.5 | 146 | 67.9 | 68 | 31.6 | 215 | 100 |
|  | **all** | 1 | 0.5 | 148 | 68.2 | 68 | 31.3 | 217 | 100 |
| **3** | **no** | 0 | 0 | 0 | 0 | 0 | 0 | 0 | 0 |
|  | **yes** | 2 | 1.0 | 131 | 64.5 | 70 | 34.5 | 203 | 100 |
|  | **all** | 2 | 1.0 | 131 | 64.5 | 70 | 34.5 | 203 | 100 |
| **4** | **no** | 0 | 0 | 0 | 0.0 | 0 | 0 | 0 | 0 |
|  | **yes** | 1 | 0.6 | 89 | 57.4 | 65 | 41.9 | 155 | 100 |
|  | **all** | 1 | 0.6 | 89 | 57.4 | 65 | 41.9 | 155 | 100 |

Supplemental Table 2: Bacterial colonization in the course of the study

| **Nasopharyngeal swap samples** | **Visit 1** | | **Visit 2** | | **Visit 3** | | **Visit 4** | |
| --- | --- | --- | --- | --- | --- | --- | --- | --- |
|  | **n** | **%** | **n** | **%** | **n** | **%** | **n** | **%** |
| **one strain** | n=48 | | n=80 | | n=56 | | n=56 | |
| ***S. pneumoniae*** | 23 | 47.9 | 35 | 43.8 | 19 | 33.9 | 17 | 30.4 |
| ***M. catarrhalis*** | 15 | 31.3 | 31 | 38.8 | 21 | 37.5 | 16 | 28.6 |
| ***H. influenzae*** | 9 | 18.8 | 14 | 17.5 | 15 | 26.8 | 21 | 37.5 |
| ***S. pyogenes*** | 1 | 2.1 | 0 | 0.0 | 1 | 1.8 | 2 | 3.6 |
| **two strains** | n=13 | | n=34 | | n=47 | | n=41 | |
| ***M. catarrhalis / S. pneumoniae*** | 4 | 30.8 | 17 | 50.0 | 26 | 55.3 | 11 | 26.8 |
| ***H. influenzae /  S. pneumoniae*** | 5 | 38.5 | 10 | 29.4 | 12 | 25.5 | 26 | 63.4 |
| ***H. influenzae /  M. catarrhalis*** | 3 | 23.1 | 6 | 17.6 | 6 | 12.8 | 2 | 4.9 |
| ***H. influenzae /  S. pyogenes*** | 1 | 7.7 | 0 | 0 | 1 | 2.1 | 2 | 4.9 |
| ***H. influenzae /  H. influenzae*** | 0 | 0 | 0 | 0 | 1 | 2.1 | 0 | 0 |
| ***M. catarrhalis /  S. pyogenes*** | 0 | 0 | 0 | 0 | 1 | 2.1 | 0 | 0 |
| ***S. pneumoniae /  S. pyogenes*** | 0 | 0 | 1 | 2.9 | 0 | 0 | 0 | 0 |
| **three strains** | n=2 | | n=5 | | n=13 | | n=11 | |
| ***H. influenzae /  M. catarrhalis/  S. pneumoniae*** | 2 | 100.0 | 4 | 80.0 | 13 | 100 | 7 | 63.6 |
| ***H. influenzae (I) /  H. influenzae (II) / S. pneumoniae*** | 0 | 0 | 1 | 20.0 | 0 | 0 | 1 | 9.1 |
| ***H. influenzae /  S. pneumoniae /  S. pyogenes*** | 0 | 0 | 0 | 0 | 0 | 0 | 1 | 9.1 |
| ***M. catarrhalis /  S. pneumoniae /  S. pyogenes*** | 0 | 0 | 0 | 0 | 0 | 0 | 2 | 18.2 |

Supplemental Table 3: Logistic model for incidence of *S. pneumoniae* found at visits

| **Visit** | **Features** | **Odds-Ratio (95%CI)** |
| --- | --- | --- |
| **Visit 2** | **relatives** | 2.15 (1.50-3.09) |
|  | **domestic tobacco smoke exposed no vs. yes** | 1.13 (0.58-2.20) |
|  | **age** | 0.93 (0.86-1) |
|  | **booster no vs. yes** | 3.14 (0.62-15.9) |
|  | **gender female vs. male** | 0.79 (0.42-1.47) |
| **Visit 3** | **relatives** | 1.38 (0.99-1.92) |
|  | **domestic tobacco smoke exposed no vs. yes** | 1.30 (0.67-2.50) |
|  | **age** | 0.96 (0.93-0.98) |
|  | **booster no vs. yes** | 0.77 (0.34-1.75) |
|  | **gender female vs. male** | 0.65 (0.36-1.19) |

Supplemental Table 4: Dynamics of colonizing pneumococcal serotypes and multi locus sequence types (MLST). Orange: PCV7 serotypes; blue: additional PCV13 serotypes; yellow: serotypes solely included in PPV23; no visit: subject didn’t show up for scheduled visit, n.d.: MLST was not done. Empty cells indicate no carriage of pneumococci.

| **Subject** | **Visit 1** | | **Visit 2** | | **Visit 3** | | **Visit 4** | |
| --- | --- | --- | --- | --- | --- | --- | --- | --- |
|  | **Serotype** | **MLST** | **Serotype** | **MLST** | **Serotype** | **MLST** | **Serotype** | **MLST** |
| 0508 | 6A | 8433 | 6A | 8436 | 23B | 9409 | 35B | 452 |
| 0319 | 9N | 66 | 19A | 994 | 23B | 439 | 11A | 62 |
| 0203 | 11A | 8428 | 23B | 439 | 6C | 1692 | 11A | 393 |
| 0202 | 11A | 8428 | 23B | 439 | 6C | 1692 | 15C | 2704 |
| 0235 | 15C | 199 | 15B | 199 | 3 | 1377 | 3 | 1377 |
| 0211 | 23B | 439 | 16F | 30 | 15C | 199 | 31 | 10468 |
| 0107 | 6B | 146 | 22F | 433 | 11A | 62 |  |  |
| 0221 | 9N | 66 | 9N | 66 | NT | 66 |  |  |
| 0112 | 9N | 66 | 9N | 66 | 33F | 100 |  |  |
| 0415 | 11A | 62 | 11A | 62 | 35F | n.d. |  |  |
| 0407 | 11A | 62 | 15B | 199 | 15A | 473 |  |  |
| 0421 | 11A | 62 | 15B | 1262 | 15C | 1262 |  |  |
| 0116 | 15B | n.d. | 35C | 433 | 15C | 199 | no visit | |
| 0607 | 19F | 242 | 6A | 8436 | 35F | 1635 |  |  |
| 0447 | 34 | 4083 | 22F | 433 | 19A | 2404 |  |  |
| 0126 | 22F | 433 | 11A | 62 |  |  | 35B | 452 |
| 0619 | 1 | 179 | 19F | 179 |  |  | no visit | |
| 0620 | 1 | 179 | 9N | 66 |  |  |  |  |
| 0306 | 11A | 62 | 35F | 446 |  |  |  |  |
| 0234 | 11A | 62 | 7F | 191 |  |  |  |  |
| 0437 | 15B | 199 | 28F | 546 |  |  |  |  |
| 0603 | 19F | 3016 | 23F | 242 |  |  |  |  |
| 0105 | 23A | 8427 | 19A | 8430 |  |  |  |  |
| 0424 | 35F | 446 | 35F | 446 |  |  |  |  |
| 0612 | 11A | 62 |  |  | 15C | 1262 | 19A | 199 |
| 0521 | 15C | 4056 |  |  | 15C | 4056 | 15A | 473 |
| 0237 | 6A | 490 |  |  | 15C | 199 | no visit | |
| 0518 | 6A | 1143 |  |  | 3 | 180 |  |  |
| 0119 | 6B | 1692 |  |  |  |  | 23A | 438 |
| 0522 | 9N | 66 |  |  |  |  | 38 | 393 |
| 0219 | 6A | 8434 |  |  |  |  |  |  |
| 0420 | 15B | 1262 |  |  |  |  |  |  |
| 0114 | 19A | 1611 |  |  | no visit | | no visit | |
| 0313 | 38 | n.d. |  |  | no visit | | no visit | |
| 0132 |  |  | 3 | 180 | 24F | n.d. | 31 | 1766 |
| 0141 |  |  | 6A | 473 | 15C | 199 | 15C | 1262 |
| 0451 |  |  | 15B | 200 | 6C | 9403 | 22F | 433 |
| 0324 |  |  | 35B | 3003 | 23F | 36 | 15B | 1262 |
| 0101 |  |  | 35F | 446 | 23A | 2958 | 19A | 1848 |
| 0301 |  |  | 35F | 1635 | 24F | 72 | 10A | 4753 |
| 0118 |  |  | 6A | 547 | 6A | 547 |  |  |
| 0507 |  |  | 6C | 176 | 6B | 176 |  |  |
| 0902 |  |  | 10A | 8441 | 10A | 8441 |  |  |
| 0903 |  |  | 10A | 8441 | 16F | 9402 |  |  |
| 0108 |  |  | 11A | 62 | 19A | 199 |  |  |
| 0446 |  |  | 11A | 62 | 19A | 3863 |  |  |
| 0140 |  |  | 11A | 1635 | 6C | 176 |  |  |
| 0450 |  |  | 15C | 3557 | 15C | 3557 |  |  |
| 0455 |  |  | 19F | 654 | 22F | 433 |  |  |
| 0302 |  |  | 23B | 439 | 21 | 193 |  |  |
| 0443 |  |  | 23F | 36 | 15C | 1262 |  |  |
| 0418 |  |  | 31 | 1994 | 23F | 9307 |  |  |
| 0321 |  |  | 35B | 452 | 35D | 452 |  |  |
| 0206 |  |  | 35F | 4849 | 3 | 180 |  |  |
| 0610 |  |  | 6A | 2557 |  |  | 23B | 2372 |
| 0204 |  |  | 8 | 53 |  |  | 9N | 66 |
| 0525 |  |  | 11A | 62 |  |  | 24F | 9870 |
| 0602 |  |  | 11A | 9310 |  |  | 28F | 546 |
| 0212 |  |  | 15B | 199 |  |  | 23B | 563 |
| 0460 |  |  | 23A | 6744 |  |  | 15C | 199 |
| 0303 |  |  | 23F | 242 |  |  | 35B | 558 |
| 0901 |  |  | 6A | 813 |  |  |  |  |
| 0412 |  |  | 6C | 1379 |  |  |  |  |
| 0109 |  |  | 9V | 162 |  |  |  |  |
| 0316 |  |  | 10A | 1551 |  |  |  |  |
| 0432 |  |  | 11A | 8432 |  |  |  |  |
| 0617 |  |  | 11A | 62 |  |  | no visit | |
| 0236 |  |  | 15B | 199 |  |  |  |  |
| 0428 |  |  | 15C | 199 |  |  |  |  |
| 0456 |  |  | 15C | 199 |  |  |  |  |
| 0705 |  |  | 18C | n.d. |  |  |  |  |
| 0702 |  |  | 23F | 6284 |  |  |  |  |
| 0142 |  |  | 19A | 319 |  |  |  |  |
| 0201 |  |  | 19A | 1611 |  |  |  |  |
| 0327 |  |  | 19A | 994 |  |  |  |  |
| 0218 |  |  | 28A | 494 |  |  |  |  |
| 0501 |  |  | 35F | 446 | no visit | | no visit | |
| 0801 |  |  | 35F | 1635 |  |  |  |  |
| 0215 |  |  |  |  | 6A | 490 | 21 | 9869 |
| 0404 |  |  |  |  | 6C | 1692 | 24F | 72 |
| 0222 |  |  |  |  | 11A | 62 | 11A | 62 |
| 0438 |  |  |  |  | 11A | 62 | 35F | 100 |
| 0317 |  |  |  |  | 11A | 62 | NT | 449 |
| 0318 |  |  |  |  | 15B | 3557 | 11A | 62 |
| 0113 |  |  |  |  | 15C | 199 | 9N | 66 |
| 0224 |  |  |  |  | 19A | 320 | 21 | 1877 |
| 0213 |  |  |  |  | 19F | 177 | 33F | 2705 |
| 0208 |  |  |  |  | 22F | 433 | 3 | 180 |
| 0138 |  |  |  |  | 23A | 4110 | 11A | 156 |
| 0304 |  |  |  |  | 23A | 438 | NT | 449 |
| 0710 |  |  |  |  | 23B | 9410 | 11A | 62 |
| 0622 |  |  |  |  | 35F | 446 | 38 | 393 |
| 0103 |  |  |  |  | 19A | 193 | no visit | |
| 0803 |  |  |  |  | 6A | 681 |  |  |
| 0614 |  |  |  |  | 6C | 1692 |  |  |
| 0422 |  |  |  |  | 6C | 1692 |  |  |
| 0445 |  |  |  |  | 11A | 62 |  |  |
| 0453 |  |  |  |  | 14 | 9 |  |  |
| 0226 |  |  |  |  | 15A | 5139 |  |  |
| 0505 |  |  |  |  | 15B | 9407 |  |  |
| 0129 |  |  |  |  | 17F | 392 |  |  |
| 0223 |  |  |  |  | 19A | 320 |  |  |
| 0431 |  |  |  |  | 22F | 433 |  |  |
| 0448 |  |  |  |  | 23A | 42 |  |  |
| 0102 |  |  |  |  | 23A | 190 |  |  |
| 0416 |  |  |  |  | 33F | 100 |  |  |
| 0110 |  |  |  |  | 35B | 9406 |  |  |
| 0423 |  |  |  |  | 35F | 446 |  |  |
| 0315 |  |  |  |  | NT | 344 |  |  |
| 0111 |  |  |  |  |  |  | 3 | 180 |
| 0329 |  |  |  |  |  |  | 3 | 180 |
| 0512 |  |  |  |  |  |  | 8 | 53 |
| 0435 |  |  |  |  |  |  | 10B | 473 |
| 0216 |  |  |  |  |  |  | 11A | 62 |
| 0233 |  |  |  |  |  |  | 15A | 63 |
| 0314 |  |  |  |  |  |  | 21 | 432 |
| 0322 |  |  |  |  |  |  | 22F | 819 |
| 0323 |  |  |  |  |  |  | 22F | 433 |
| 0524 |  |  |  |  |  |  | 22F | 433 |
| 0325 |  |  |  |  |  |  | 23A | 438 |
| 0417 |  |  |  |  |  |  | 23A | 42 |
| 0519 |  |  |  |  |  |  | 23A | 2319 |
| 0712 |  |  |  |  |  |  | 23A | 2958 |
| 0123 |  |  |  |  |  |  | 24F | 177 |
| 0320 |  |  |  |  |  |  | 24F | 72 |
| 0704 |  |  |  |  |  |  | 24F | 162 |
| 0711 |  |  |  |  |  |  | 24F | 162 |
| 0220 |  |  |  |  |  |  | 31 | 1766 |
| 0136 |  |  |  |  |  |  | 33F | 100 |
| 0309 |  |  |  |  |  |  | 33F | 673 |
| 0502 |  |  |  |  |  |  | 34 | 547 |
| 0440 |  |  |  |  |  |  | 35F | 1635 |
| 0510 |  |  |  |  |  |  | 35F | 446 |
| 0115 |  |  |  |  |  |  | NT | 448 |
| 0458 |  |  |  |  |  |  | NT | 1229 |
| 0459 |  |  |  |  |  |  | NT | 1229 |
|  | **34** |  | **68** |  | **70** |  | **65** |  |

**Supplemental Table 5:** Multi locus sequence types of *S. pneumonia* isolates. n.d.: MLST was not done.

| **Subject** | **Visit Nr** | **Serotype** | **Sequence type** | ***aroE*** | ***gdh*** | ***gki*** | ***recP*** | ***Spi*** | ***xpt*** | ***ddl*** |
| --- | --- | --- | --- | --- | --- | --- | --- | --- | --- | --- |
| 0202 | 1 | 11A | 8428 | 2 | 5 | 14 | 12 | 16 | 3 | 14 |
| 0202 | 2 | 23B | 439 | 1 | 8 | 9 | 2 | 6 | 4 | 6 |
| 0202 | 3 | 6C | 1692 | 1 | 5 | 7 | 12 | 17 | 158 | 14 |
| 0202 | 4 | 15C | 2704 | 8 | 13 | 14 | 10 | 17 | 4 | 14 |
| 0203 | 1 | 11A | 8428 | 2 | 5 | 14 | 12 | 16 | 3 | 14 |
| 0203 | 2 | 23B | 439 | 1 | 8 | 9 | 2 | 6 | 4 | 6 |
| 0203 | 3 | 6C | 1692 | 1 | 5 | 7 | 12 | 17 | 158 | 14 |
| 0203 | 4 | 11A | 393 | 10 | 43 | 41 | 18 | 13 | 49 | 6 |
| 0211 | 1 | 23B | 439 | 1 | 8 | 9 | 2 | 6 | 4 | 6 |
| 0211 | 2 | 16F | 30 | 1 | 5 | 27 | 20 | 1 | 1 | 1 |
| 0211 | 3 | 15C | 199 | 8 | 13 | 14 | 4 | 17 | 4 | 14 |
| 0211 | 4 | 31 | 10468 | 5 | 5 | 29 | 1 | 46 | 14 | 18 |
| 0235 | 1 | 15C | 199 | 8 | 13 | 14 | 4 | 17 | 4 | 14 |
| 0235 | 2 | 15B | 199 | 8 | 13 | 14 | 4 | 17 | 4 | 14 |
| 0235 | 3 | 3 | 1377 | 13 | 84 | 15 | 14 | 10 | 16 | 19 |
| 0235 | 4 | 3 | 1377 | 13 | 84 | 15 | 14 | 10 | 16 | 19 |
| 0319 | 1 | 9N | 66 | 2 | 8 | 2 | 4 | 6 | 1 | 1 |
| 0319 | 2 | 19A | 994 | 5 | 5 | 62 | 5 | 6 | 11 | 14 |
| 0319 | 3 | 23B | 439 | 1 | 8 | 9 | 2 | 6 | 4 | 6 |
| 0319 | 4 | 11A | 62 | 2 | 5 | 29 | 12 | 16 | 3 | 14 |
| 0508 | 1 | 6A | 8433 | 2 | 5 | 9 | 1 | 168 | 19 | 14 |
| 0508 | 2 | 6A | 8436 | 2 | 272 | 9 | 1 | 6 | 19 | 14 |
| 0508 | 3 | 23B | 9409 | 1 | 8 | 9 | 2 | 6 | 554 | 6 |
| 0508 | 4 | 35B | 452 | 7 | 9 | 19 | 1 | 14 | 48 | 14 |
| 0101 | 2 | 35F | 446 | 5 | 7 | 4 | 19 | 10 | 40 | 27 |
| 0101 | 3 | 23A | 2958 | 1 | 13 | 9 | 9 | 6 | 4 | 6 |
| 0101 | 4 | 19A | 1848 | 10 | 8 | 30 | 35 | 6 | 1 | 9 |
| 0107 | 1 | 6B | 146 | 7 | 6 | 1 | 2 | 6 | 15 | 14 |
| 0107 | 2 | 22F | 433 | 1 | 1 | 4 | 1 | 18 | 58 | 17 |
| 0107 | 3 | 11A | 62 | 2 | 5 | 29 | 12 | 16 | 3 | 14 |
| 0112 | 1 | 9N | 66 | 2 | 8 | 2 | 4 | 6 | 1 | 1 |
| 0112 | 2 | 9N | 66 | 2 | 8 | 2 | 4 | 6 | 1 | 1 |
| 0112 | 3 | 33F | 100 | 5 | 12 | 29 | 12 | 9 | 39 | 18 |
| 0116 | 1 | 15B | n.d. |  |  |  |  |  |  |  |
| 0116 | 2 | 35C | 433 | 1 | 1 | 4 | 1 | 18 | 58 | 17 |
| 0116 | 3 | 15C | 199 | 8 | 13 | 14 | 4 | 17 | 4 | 14 |
| 0126 | 1 | 22F | 433 | 1 | 1 | 4 | 1 | 18 | 58 | 17 |
| 0126 | 2 | 11A | 62 | 2 | 5 | 29 | 12 | 16 | 3 | 14 |
| 0126 | 4 | 35B | 452 | 7 | 9 | 19 | 1 | 14 | 48 | 14 |
| 0132 | 2 | 3 | 180 | 7 | 15 | 2 | 10 | 6 | 1 | 22 |
| 0132 | 3 | 24F | n.d. |  |  |  |  |  |  |  |
| 0132 | 4 | 31 | 1766 | 1 | 5 | 29 | 1 | 46 | 14 | 18 |
| 0141 | 2 | 6A | 473 | 7 | 25 | 4 | 4 | 15 | 20 | 28 |
| 0141 | 3 | 15C | 199 | 8 | 13 | 14 | 4 | 17 | 4 | 14 |
| 0141 | 4 | 15C | 1262 | 7 | 41 | 2 | 6 | 10 | 26 | 1 |
| 0221 | 1 | 9N | 66 | 2 | 8 | 2 | 4 | 6 | 1 | 1 |
| 0221 | 2 | 9N | 66 | 2 | 8 | 2 | 4 | 6 | 1 | 1 |
| , |  |  |  |  |  |  |  |  |  |  |
| 0221 | 3 | NT | 66 | 2 | 8 | 2 | 4 | 6 | 1 | 1 |
| 0301 | 2 | 35F | 1635 | 10 | 7 | 4 | 19 | 10 | 40 | 27 |
| 0301 | 3 | 24F | 72 | 2 | 13 | 2 | 4 | 9 | 4 | 1 |
| 0301 | 4 | 10A | 4753 | 5 | 241 | 4 | 2 | 10 | 1 | 27 |
| 0324 | 2 | 35B | 3003 | 8 | 13 | 4 | 8 | 162 | 22 | 34 |
| 0324 | 3 | 23F | 36 | 1 | 8 | 4 | 1 | 1 | 4 | 6 |
| 0324 | 4 | 15B | 1262 | 7 | 41 | 2 | 6 | 10 | 26 | 1 |
| 0407 | 1 | 11A | 62 | 2 | 5 | 29 | 12 | 16 | 3 | 14 |
| 0407 | 2 | 15B | 199 | 8 | 13 | 14 | 4 | 17 | 4 | 14 |
| 0407 | 3 | 15A | 473 | 7 | 25 | 4 | 4 | 15 | 20 | 28 |
| 0415 | 1 | 11A | 62 | 2 | 5 | 29 | 12 | 16 | 3 | 14 |
| 0415 | 2 | 11A | 62 | 2 | 5 | 29 | 12 | 16 | 3 | 14 |
| 0415 | 3 | 35F | n.d. |  |  |  |  |  |  |  |
| 0421 | 1 | 11A | 62 | 2 | 5 | 29 | 12 | 16 | 3 | 14 |
| 0421 | 2 | 15B | 1262 | 7 | 41 | 2 | 6 | 10 | 26 | 1 |
| 0421 | 3 | 15C | 1262 | 7 | 41 | 2 | 6 | 10 | 26 | 1 |
| 0447 | 1 | 34 | 4083 | 7 | 5 | 53 | 5 | 42 | 1 | 79 |
| 0447 | 2 | 22F | 433 | 1 | 1 | 4 | 1 | 18 | 58 | 17 |
| 0447 | 3 | 19A | 2404 | 7 | 8 | 9 | 9 | 6 | 4 | 6 |
| 0451 | 2 | 15B | 200 | 8 | 13 | 14 | 4 | 1 | 4 | 14 |
| 0451 | 3 | 6C | 9403 | 1 | 8 | 15 | 44 | 17 | 7 | 14 |
| 0451 | 4 | 22F | 433 | 1 | 1 | 4 | 1 | 18 | 58 | 17 |
| 0521 | 1 | 15C | 4056 | 8 | 13 | 14 | 4 | 17 | 51 | 14 |
| 0521 | 3 | 15C | 4056 | 8 | 13 | 14 | 4 | 17 | 51 | 14 |
| 0521 | 4 | 15A | 473 | 7 | 25 | 4 | 4 | 15 | 20 | 28 |
| 0607 | 1 | 19F | 242 | 15 | 29 | 4 | 21 | 30 | 1 | 14 |
| 0607 | 2 | 6A | 8436 | 2 | 272 | 9 | 1 | 6 | 19 | 14 |
| 0607 | 3 | 35F | 1635 | 10 | 7 | 4 | 19 | 10 | 40 | 27 |
| 0612 | 1 | 11A | 62 | 2 | 5 | 29 | 12 | 16 | 3 | 14 |
| 0612 | 3 | 15C | 1262 | 7 | 41 | 2 | 6 | 10 | 26 | 1 |
| 0612 | 4 | 19A | 199 | 8 | 13 | 14 | 4 | 17 | 4 | 14 |
| 0622 | 3 | 35F | 446 | 5 | 7 | 4 | 19 | 10 | 40 | 27 |
| 0622 | 3 | 35F | 446 | 5 | 7 | 4 | 19 | 10 | 40 | 27 |
| 0622 | 4 | 38 | 393 | 10 | 43 | 41 | 18 | 13 | 49 | 6 |
| 0105 | 1 | 23A | 8427 | 1 | 8 | 9 | 9 | 5 | 4 | 6 |
| 0105 | 2 | 19A | 8430 | 16 | 43 | 4 | 8 | 14 | 12 | 29 |
| 0108 | 2 | 11A | 62 | 2 | 5 | 29 | 12 | 16 | 3 | 14 |
| 0108 | 3 | 19A | 199 | 8 | 13 | 14 | 4 | 17 | 4 | 14 |
| 0113 | 3 | 15C | 199 | 8 | 13 | 14 | 4 | 17 | 4 | 14 |
| 0113 | 4 | 9N | 66 | 2 | 8 | 2 | 4 | 6 | 1 | 1 |
| 0118 | 2 | 6A | 547 | 5 | 5 | 6 | 1 | 9 | 10 | 14 |
| 0118 | 3 | 6A | 547 | 5 | 5 | 6 | 1 | 9 | 10 | 14 |
| 0119 | 1 | 6B | 1692 | 1 | 5 | 7 | 12 | 17 | 158 | 14 |
| 0119 | 4 | 23A | 438 | 1 | 5 | 9 | 9 | 6 | 4 | 6 |
| 0138 | 3 | 23A | 4110 | 1 | 1 | 4 | 1 | 18 | 58 | 332 |
| 0138 | 4 | 11A | 156 | 7 | 11 | 10 | 1 | 6 | 8 | 1 |
| 0140 | 2 | 11A | 1635 | 10 | 7 | 4 | 19 | 10 | 40 | 27 |
| 0140 | 3 | 6C | 176 | 7 | 13 | 8 | 6 | 10 | 6 | 14 |
| 0204 | 2 | 8 | 53 | 2 | 5 | 1 | 11 | 16 | 3 | 14 |
| 0204 | 4 | 9N | 66 | 2 | 8 | 2 | 4 | 6 | 1 | 1 |
| 0206 | 2 | 35F | 4849 | 5 | 140 | 4 | 19 | 10 | 40 | 27 |
| 0206 | 3 | 3 | 180 | 7 | 15 | 2 | 10 | 6 | 1 | 22 |
| 0208 | 3 | 22F | 433 | 1 | 1 | 4 | 1 | 18 | 58 | 17 |
| 0208 | 4 | 3 | 180 | 7 | 15 | 2 | 10 | 6 | 1 | 22 |
| 0212 | 2 | 15B | 199 | 8 | 13 | 14 | 4 | 17 | 4 | 14 |
| 0212 | 4 | 23B | 563 | 18 | 13 | 8 | 6 | 6 | 6 | 8 |
| 0213 | 3 | 19F | 177 | 7 | 14 | 4 | 12 | 1 | 1 | 14 |
| 0213 | 4 | 33F | 2705 | 5 | 12 | 29 | 16 | 9 | 39 | 1 |
| 0215 | 3 | 6A | 490 | 2 | 13 | 9 | 1 | 6 | 19 | 14 |
| 0215 | 4 | 21 | 9869 | 10 | 8 | 4 | 35 | 6 | 182 | 14 |
| 0222 | 3 | 11A | 62 | 2 | 5 | 29 | 12 | 16 | 3 | 14 |
| 0222 | 4 | 11A | 62 | 2 | 5 | 29 | 12 | 16 | 3 | 14 |
| 0224 | 3 | 19A | 320 | 4 | 16 | 19 | 15 | 6 | 20 | 1 |
| 0224 | 4 | 21 | 1877 | 10 | 10 | 41 | 16 | 1 | 26 | 1 |
| 0234 | 1 | 11A | 62 | 2 | 5 | 29 | 12 | 16 | 3 | 14 |
| 0234 | 2 | 7F | 191 | 8 | 9 | 2 | 1 | 6 | 1 | 17 |
| 0237 | 1 | 6A | 490 | 2 | 13 | 9 | 1 | 6 | 19 | 14 |
| 0237 | 3 | 15C | 199 | 8 | 13 | 14 | 4 | 17 | 4 | 14 |
| 0302 | 2 | 23B | 439 | 1 | 8 | 9 | 2 | 6 | 4 | 6 |
| 0302 | 3 | 21 | 193 | 8 | 10 | 2 | 16 | 1 | 26 | 1 |
| 0303 | 2 | 23F | 242 | 15 | 29 | 4 | 21 | 30 | 1 | 14 |
| 0303 | 4 | 35B | 558 | 18 | 12 | 4 | 44 | 14 | 77 | 97 |
| 0304 | 3 | 23A | 438 | 1 | 5 | 9 | 9 | 6 | 4 | 6 |
| 0304 | 4 | NT | 449 | 8 | 37 | 9 | 29 | 2 | 47 | 5 |
| 0306 | 1 | 11A | 62 | 2 | 5 | 29 | 12 | 16 | 3 | 14 |
| 0306 | 2 | 35F | 446 | 5 | 7 | 4 | 19 | 10 | 40 | 27 |
| 0317 | 3 | 11A | 62 | 2 | 5 | 29 | 12 | 16 | 3 | 14 |
| 0317 | 4 | NT | 449 | 8 | 37 | 9 | 29 | 2 | 47 | 5 |
| 0318 | 3 | 15B | 3557 | 7 | 41 | 47 | 16 | 6 | 14 | 17 |
| 0318 | 4 | 11A | 62 | 2 | 5 | 29 | 12 | 16 | 3 | 14 |
| 0321 | 2 | 35B | 452 | 7 | 9 | 19 | 1 | 14 | 48 | 14 |
| 0321 | 3 | 35D | 452 | 7 | 9 | 19 | 1 | 14 | 48 | 14 |
| 0404 | 3 | 6C | 1692 | 1 | 5 | 7 | 12 | 17 | 158 | 14 |
| 0404 | 4 | 24F | 72 | 2 | 13 | 2 | 4 | 9 | 4 | 1 |
| 0418 | 2 | 31 | 1994 | 1 | 2 | 29 | 1 | 111 | 14 | 18 |
| 0418 | 3 | 23F | 9307 | 7 | 5 | 1 | 1 | 397 | 31 | 14 |
| 0424 | 1 | 35F | 446 | 5 | 7 | 4 | 19 | 10 | 40 | 27 |
| 0424 | 2 | 35F | 446 | 5 | 7 | 4 | 19 | 10 | 40 | 27 |
| 0437 | 1 | 15B | 199 | 8 | 13 | 14 | 4 | 17 | 4 | 14 |
| 0437 | 2 | 28F | 546 | 16 | 44 | 1 | 16 | 9 | 11 | 17 |
| 0438 | 3 | 11A | 62 | 2 | 5 | 29 | 12 | 16 | 3 | 14 |
| 0438 | 4 | 35F | 100 | 5 | 12 | 29 | 12 | 9 | 39 | 18 |
| 0443 | 2 | 23F | 36 | 1 | 8 | 4 | 1 | 1 | 4 | 6 |
| 0443 | 3 | 15C | 1262 | 7 | 41 | 2 | 6 | 10 | 26 | 1 |
| 0446 | 2 | 11A | 62 | 2 | 5 | 29 | 12 | 16 | 3 | 14 |
| 0446 | 3 | 19A | 3863 | 8 | 10 | 211 | 16 | 1 | 26 | 1 |
| 0450 | 2 | 15C | 3557 | 7 | 41 | 47 | 16 | 6 | 14 | 17 |
| 0450 | 3 | 15C | 3557 | 7 | 41 | 47 | 16 | 6 | 14 | 17 |
| 0455 | 2 | 19F | 654 | 18 | 2 | 22 | 16 | 9 | 23 | 14 |
| 0455 | 3 | 22F | 433 | 1 | 1 | 4 | 1 | 18 | 58 | 17 |
| 0460 | 2 | 23A | 6744 | 1 | 60 | 9 | 9 | 6 | 4 | 6 |
| 0460 | 4 | 15C | 199 | 8 | 13 | 14 | 4 | 17 | 4 | 14 |
| 0507 | 2 | 6C | 176 | 7 | 13 | 8 | 6 | 10 | 6 | 14 |
| 0507 | 3 | 6B | 176 | 7 | 13 | 8 | 6 | 10 | 6 | 14 |
| 0518 | 1 | 6A | 1143 | 7 | 25 | 4 | 1 | 15 | 1 | 28 |
| 0518 | 3 | 3 | 180 | 7 | 15 | 2 | 10 | 6 | 1 | 22 |
| 0522 | 1 | 9N | 66 | 2 | 8 | 2 | 4 | 6 | 1 | 1 |
| 0522 | 4 | 38 | 393 | 10 | 43 | 41 | 18 | 13 | 49 | 6 |
| 0525 | 2 | 11A | 62 | 2 | 5 | 29 | 12 | 16 | 3 | 14 |
| 0525 | 4 | 24F | 9870 | 7 | 11 | 10 | 1 | 6 | 8 | 20 |
| 0602 | 2 | 11A | 9310 | 2 | 16 | 29 | 18 | 42 | 546 | 18 |
| 0602 | 4 | 28F | 546 | 16 | 44 | 1 | 16 | 9 | 11 | 17 |
| 0603 | 1 | 19F | 3016 | 1 | 5 | 4 | 12 | 5 | 3 | 230 |
| 0603 | 2 | 23F | 242 | 15 | 29 | 4 | 21 | 30 | 1 | 14 |
| 0610 | 2 | 6A | 2557 | 5 | 5 | 4 | 1 | 9 | 1 | 19 |
| 0610 | 4 | 23B | 2372 | 18 | 13 | 8 | 6 | 3 | 6 | 46 |
| 0619 | 1 | 1 | 179 | 7 | 14 | 40 | 12 | 1 | 1 | 14 |
| 0619 | 2 | 19F | 179 | 7 | 14 | 40 | 12 | 1 | 1 | 14 |
| 0620 | 1 | 1 | 179 | 7 | 14 | 40 | 12 | 1 | 1 | 14 |
| 0620 | 2 | 9N | 66 | 2 | 8 | 2 | 4 | 6 | 1 | 1 |
| 0710 | 3 | 23B | 9410 | 1 | 8 | 9 | 2 | 6 | 555 | 6 |
| 0710 | 4 | 11A | 62 | 2 | 5 | 29 | 12 | 16 | 3 | 14 |
| 0902 | 2 | 10A | 8441 | 5 | 7 | 4 | 2 | 10 | 12 | 6 |
| 0902 | 3 | 10A | 8441 | 5 | 7 | 4 | 2 | 10 | 12 | 6 |
| 0903 | 2 | 10A | 8441 | 5 | 7 | 4 | 2 | 10 | 12 | 6 |
| 0903 | 3 | 16F | 9402 | 7 | 8 | 8 | 66 | 9 | 103 | 245 |
| 0102 | 3 | 23A | 190 | 8 | 8 | 9 | 9 | 6 | 4 | 6 |
| 0103 | 3 | 19A | 193 | 8 | 10 | 2 | 16 | 1 | 26 | 1 |
| 0109 | 2 | 9V | 162 | 7 | 11 | 10 | 1 | 6 | 8 | 14 |
| 0110 | 3 | 35B | 9406 | 8 | 407 | 4 | 8 | 162 | 22 | 34 |
| 0111 | 4 | 3 | 180 | 7 | 15 | 2 | 10 | 6 | 1 | 22 |
| 0114 | 1 | 19A | 1611 | 2 | 19 | 2 | 17 | 15 | 22 | 14 |
| 0115 | 4 | NT | 448 | 8 | 5 | 2 | 27 | 2 | 11 | 71 |
| 0123 | 4 | 24F | 177 | 7 | 14 | 4 | 12 | 1 | 1 | 14 |
| 0129 | 3 | 17F | 392 | 7 | 5 | 1 | 1 | 6 | 31 | 14 |
| 0136 | 4 | 33F | 100 | 5 | 12 | 29 | 12 | 9 | 39 | 18 |
| 0142 | 2 | 19A | 319 | 12 | 19 | 2 | 17 | 6 | 22 | 9 |
| 0201 | 2 | 19A | 1611 | 2 | 19 | 2 | 17 | 15 | 22 | 14 |
| 0216 | 4 | 11A | 62 | 2 | 5 | 29 | 12 | 16 | 3 | 14 |
| 0218 | 2 | 28A | 494 | 16 | 44 | 1 | 16 | 9 | 70 | 17 |
| 0219 | 1 | 6A | 8434 | 2 | 13 | 9 | 11 | 6 | 19 | 14 |
| 0220 | 4 | 31 | 1766 | 1 | 5 | 29 | 1 | 46 | 14 | 18 |
| 0223 | 3 | 19A | 320 | 4 | 16 | 19 | 15 | 6 | 20 | 1 |
| 0226 | 3 | 15A | 5139 | 8 | 5 | 2 | 6 | 1 | 26 | 1 |
| 0233 | 4 | 15A | 63 | 2 | 5 | 36 | 12 | 17 | 21 | 14 |
| 0236 | 2 | 15B | 199 | 8 | 13 | 14 | 4 | 17 | 4 | 14 |
| 0309 | 4 | 33F | 673 | 2 | 5 | 29 | 16 | 42 | 3 | 18 |
| 0310 | 1 | 19A | 5912 | 10 | 19 | 2 | 17 | 6 | 22 | 14 |
| 0313 | 1 | 38 | n.d. |  |  |  |  |  |  |  |
| 0314 | 4 | 21 | 432 | 10 | 8 | 4 | 35 | 6 | 1 | 14 |
| 0315 | 3 | NT | 344 | 8 | 37 | 9 | 29 | 2 | 12 | 53 |
| 0316 | 2 | 10A | 1551 | 5 | 7 | 4 | 2 | 10 | 1 | 6 |
| 0320 | 4 | 24F | 72 | 2 | 13 | 2 | 4 | 9 | 4 | 1 |
| 0322 | 4 | 22F | 819 | 1 | 1 | 4 | 1 | 18 | 58 | 18 |
| 0323 | 4 | 22F | 433 | 1 | 1 | 4 | 1 | 18 | 58 | 17 |
| 0325 | 4 | 23A | 438 | 1 | 5 | 9 | 9 | 6 | 4 | 6 |
| 0327 | 2 | 19A | 994 | 5 | 5 | 62 | 5 | 6 | 11 | 14 |
| 0329 | 4 | 3 | 180 | 7 | 15 | 2 | 10 | 6 | 1 | 22 |
| 0412 | 2 | 6C | 1379 | 1 | 5 | 9 | 12 | 94 | 28 | 20 |
| 0416 | 3 | 33F | 100 | 5 | 12 | 29 | 12 | 9 | 39 | 18 |
| 0417 | 4 | 23A | 42 | 1 | 8 | 9 | 9 | 6 | 4 | 6 |
| 0420 | 1 | 15B | 1262 | 7 | 41 | 2 | 6 | 10 | 26 | 1 |
| 0422 | 3 | 6C | 1692 | 1 | 5 | 7 | 12 | 17 | 158 | 14 |
| 0423 | 3 | 35F | 446 | 5 | 7 | 4 | 19 | 10 | 40 | 27 |
| 0428 | 2 | 15C | 199 | 8 | 13 | 14 | 4 | 17 | 4 | 14 |
| 0431 | 3 | 22F | 433 | 1 | 1 | 4 | 1 | 18 | 58 | 17 |
| 0432 | 2 | 11A | 8432 | 2 | 272 | 29 | 12 | 16 | 3 | 14 |
| 0435 | 4 | 10B | 473 | 7 | 25 | 4 | 4 | 15 | 20 | 28 |
| 0440 | 4 | 35F | 1635 | 10 | 7 | 4 | 19 | 10 | 40 | 27 |
| 0445 | 3 | 11A | 62 | 2 | 5 | 29 | 12 | 16 | 3 | 14 |
| 0448 | 3 | 23A | 42 | 1 | 8 | 9 | 9 | 6 | 4 | 6 |
| 0453 | 3 | 14 | 9 | 1 | 5 | 4 | 5 | 5 | 1 | 8 |
| 0456 | 2 | 15C | 199 | 8 | 13 | 14 | 4 | 17 | 4 | 14 |
| 0458 | 4 | NT | 1229 | 8 | 5 | 2 | 27 | 2 | 136 | 71 |
| 0459 | 4 | NT | 1229 | 8 | 5 | 2 | 27 | 2 | 136 | 71 |
| 0501 | 2 | 35F | 446 | 5 | 7 | 4 | 19 | 10 | 40 | 27 |
| 0502 | 4 | 34 | 547 | 5 | 5 | 6 | 1 | 9 | 10 | 14 |
| 0505 | 3 | 15B | 9407 | 8 | 408 | 14 | 4 | 17 | 4 | 14 |
| 0510 | 4 | 35F | 446 | 5 | 7 | 4 | 19 | 10 | 40 | 27 |
| 0512 | 4 | 8 | 53 | 2 | 5 | 1 | 11 | 16 | 3 | 14 |
| 0519 | 4 | 23A | 2319 | 1 | 8 | 9 | 4 | 6 | 4 | 6 |
| 0524 | 4 | 22F | 433 | 1 | 1 | 4 | 1 | 18 | 58 | 17 |
| 0604 | 1 | 19F | 179 | 7 | 14 | 40 | 12 | 1 | 1 | 14 |
| 0605 | 1 | 23A | 179 | 7 | 14 | 40 | 12 | 1 | 1 | 14 |
| 0614 | 3 | 6C | 1692 | 1 | 5 | 7 | 12 | 17 | 158 | 14 |
| 0617 | 2 | 11A | 62 | 2 | 5 | 29 | 12 | 16 | 3 | 14 |
| 0618 | 1 | 11A | 62 | 2 | 5 | 29 | 12 | 16 | 3 | 14 |
| 0702 | 2 | 23F | 6284 | 15 | 29 | 4 | 5 | 30 | 1 | 14 |
| 0704 | 4 | 24F | 162 | 7 | 11 | 10 | 1 | 6 | 8 | 14 |
| 0705 | 2 | 18C | n.d. |  |  |  |  |  |  |  |
| 0711 | 4 | 24F | 162 | 7 | 11 | 10 | 1 | 6 | 8 | 14 |
| 0712 | 4 | 23A | 2958 | 1 | 13 | 9 | 9 | 6 | 4 | 6 |
| 0801 | 2 | 35F | 1635 | 10 | 7 | 4 | 19 | 10 | 40 | 27 |
| 0803 | 3 | 6A | 681 | 2 | 5 | 9 | 1 | 6 | 19 | 14 |
| 0901 | 2 | 6A | 813 | 7 | 25 | 4 | 5 | 15 | 20 | 28 |

Supplemental Table 6: Subjects in day-care and colonization with *S. pneumoniae*

| **Visit** | **Subject in day-care** | ***S. pneumoniae*** | | | | | | **all** | |
| --- | --- | --- | --- | --- | --- | --- | --- | --- | --- |
|  |  | **unknown** | | **negative** | | **positive** | |  |  |
|  |  | **no. of subjects** | **%** | **no. of subjects** | **%** | **no. of subjects** | **%** | **no. of subjects** | **%** |
| **1** | **no** | 4 | 1.7 | 197 | 83.8 | 34 | 14.5 | 235 | 100 |
|  | **yes** | 0 | 0 | 1 | 100 | 0 | 0 | 1 | 100 |
| **2** | **no** | 1 | 0.5 | 143 | 67.5 | 68 | 32.1 | 212 | 100 |
|  | **yes** | 0 | 0 | 5 | 100 | 0 | 0 | 5 | 100 |
| **3** | **no** | 1 | 0.6 | 118 | 66.3 | 59 | 33.1 | 178 | 100 |
|  | **yes** | 1 | 4.0 | 13 | 52.0 | 11 | 44.0 | 25 | 100 |
| **4** | **no** | 1 | 25.0 | 3 | 75.0 | 0 | 0 | 4 | 100 |
|  | **yes** | 0 | 0 | 86 | 57.0 | 65 | 43.0 | 151 | 100 |

**Supplemental Figure 1:** Serotype distribution (%) of subjects carrying *S. pneumoniae* with, or without *H. influenzae*.
